# Inward Flux of Lactate^-^ through Monocarboxylate Transporters Contributes to Regulatory Volume Increase in Mouse Muscle Fibres

**DOI:** 10.1371/journal.pone.0084451

**Published:** 2013-12-23

**Authors:** Michael I. Lindinger, Matthew J. Leung, Thomas J. Hawke

**Affiliations:** 1 Department of Human Health and Nutritional Sciences, University of Guelph, Guelph, Ontario, Canada; 2 Department of Pathology & Molecular Medicine, McMaster University, Hamilton, Ontario, Canada; University of Debrecen, Hungary

## Abstract

Mouse and rat skeletal muscles are capable of a regulatory volume increase (RVI) after they shrink (volume loss resultant from exposure to solutions of increased osmolarity) and that this RVI occurs mainly by a Na-K-Cl-Cotransporter (NKCC) - dependent mechanism. With high-intensity exercise, increased extracellular osmolarity is accompanied by large increases in extracellular [lactate^-^]. We hypothesized that large increases in [lactate^-^] and osmolarity augment the NKCC-dependent RVI response observed with a NaCl (or sucrose) - induced increase in osmolarity alone; a response that is dependent on lactate^-^ influx through monocarboxylate transporters (MCTs). Single mouse muscle fibres were isolated and visualized under light microscopy under varying osmolar conditions. When solution osmolarity was increased by adding NaLac by 30 or 60 mM, fibres lost significantly less volume and regained volume sooner compared to when NaCl was used. Phloretin (MCT1 inhibitor) accentuated the volume loss compared to both NaLac controls, supporting a role for MCT1 in the RVI response in the presence of elevated [lactate^-^]. Inhibition of MCT4 (with pCMBS) resulted in a volume loss, intermediate to that seen with phloretin and NaLac controls. Bumetanide (NKCC inhibitor), in combination with pCMBS, reduced the magnitude of volume loss, but volume recovery was complete. While combined phloretin-bumetanide also reduced the magnitude of the volume loss, it also largely abolished the cell volume recovery. In conclusion, RVI in skeletal muscle exposed to raised tonicity and [lactate^-^] is facilitated by inward flux of solute by NKCC- and MCT1-dependent mechanisms. This work demonstrates evidence of a RVI response in skeletal muscle that is facilitated by inward flux of solute by MCT-dependent mechanisms. These findings further expand our understanding of the capacities for skeletal muscle to volume regulate, particularly in instances of raised tonicity and lactate^-^ concentrations, as occurs with high intensity exercise.

## Introduction

High intensity exercise increases plasma and tissue extracellular osmolarity throughout the body due to simultaneous flux of solute-poor fluid into contracting muscles [1], [2], [3] and accumulation of lactate^-^ in extracellular fluids [4]. The increase in extracellular osmolarity results in a volume loss in non-contracting cells [1], [2] that aids in the defense of circulating blood volume loss during the first minutes of exercise [1]. In response to volume loss (and resultant cell shrinkage), skeletal muscle fibres have recently been shown to exhibit a regulatory volume increase (RVI) that is mediated by a bumetanide- and ouabain-sensitive ion transport process [5]. The transport system is believed to be the electro-neutral Na-K-2Cl co-transporter (NKCC) that is important in volume regulation in many cell types [6], [7].

**Figure 1:**
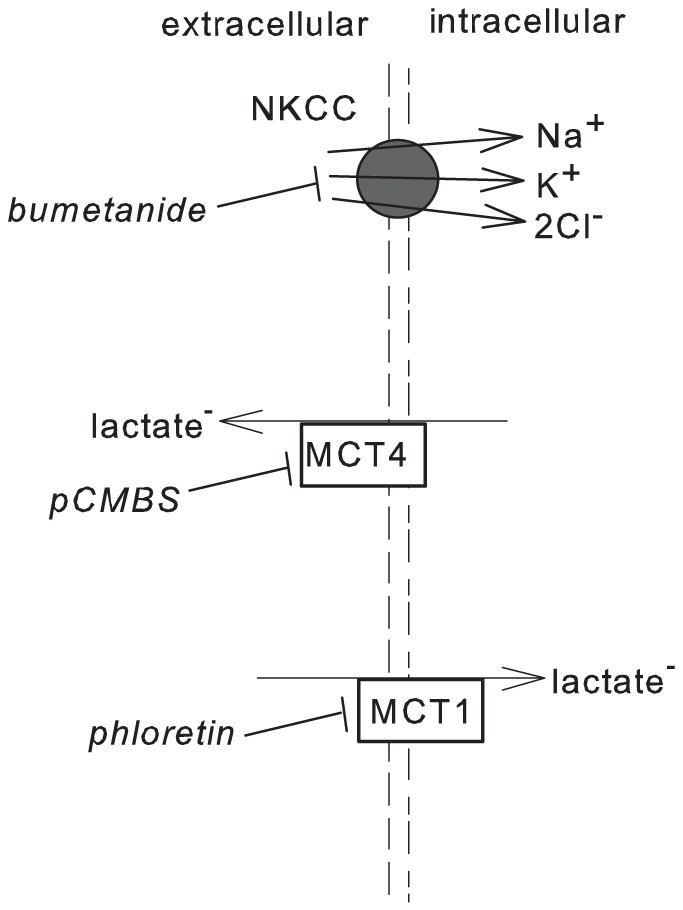
Schematic representation of known and putative ionic regulatory volume increase (RVI) mechanisms in mammalian skeletal muscle. The sodium, potassium, chloride cotransporter (NKCC) facilitates the inward flux of these three ions into cells. The NKCC can be inhibited to a large extent by 1 mM bumetanide. The two main monocarboxylate transporters (MCTs) in muscle are MCT1 and MCT4. Phloretin (1 mM) inhibits all lactate^-^ flux through MCT1 and about 90% of flux through MCT4. pCMBS inhibits all lactate^-^ flux through MCT4 and about 90% of flux through MCT1. Data presented in the present paper favour a preferential influx of lactate^-^ through MCT1 and a preferential efflux of lactate^-^ through MCT4.

Given that extracellular lactate^-^ concentration ([lactate^-^]) is increased during exercise, and because lactate^-^ is osmotically active, we hypothesized that elevated extracellular [lactate^-^] concomitant with increased extracellular osmolarity would augment the NKCC-dependent RVI (see Figure 1). In vivo, such an effect would mitigate the cell shrinkage that occurs in non-contracting muscle [1], [2] during periods of exercise. Lactate^-^ transport across skeletal muscle plasma membranes appears to occur by two primary pathways: (1) the monocarboxylate transporters (MCT) account for most (80–90%) of the flux, and (2) passive diffusion accounts for 10–20% [8]. In contrast to erythrocytes, where a chloride-bicarbonate exchanger (band 3 protein) accounts for 3–10% of net lactate^-^ transport [9], this transporter does not appear to be present in skeletal muscle [8].

The MCTs are capable of transporting lactate^-^ in both directions across the plasma membrane. The literature suggests that the direction of net lactate^-^ flux across the sarcolemma is influenced by the isoforms that are expressed [10]–[15]. While there is some variability in the literature regarding the Km (indicating the affinity for lactate^-^) for MCT1 and MCT4 in muscle and other cells [16], the evidence supports a relatively low Km (3.5 – 8.3 mM) for MCT1 [17]–[19] and a relatively high Km (25 – 34 mM) for MCT4 [18]–[20]. The low Km MCT1 is ubiquitously expressed in a variety of mammalian tissues, notably oxidative skeletal muscle and the heart [11], [12] where it primarily facilitates the inward transport of lactate^-^ [13], [15]. The MCT4 is the dominant isoform in glycolytic muscle [19], [21], and the high Km is consistent with a requirement for intracellular accumulation of lactate^-^, and retention of pyruvate, during contractile activity of muscle. MCT4 may therefore have a primary role in facilitating lactate^-^ efflux from cells during times in which lactate^-^ production exceeds pyruvate oxidation [15]. Therefore, accepting that directionality of sarcolemmal lactate^-^ transport is determined by MCT1 and MCT4 isoform expression, the use of MCT inhibitors having different affinities for the isoforms may thus be exploited. Phloretin has a K_i_ of 5 µM for MCT1 and a K_0.5_ of 30–50 µM for MCT4, while ρ-chloromercuribenzene sulphonate (pCMBS) has a K_i_ for MCT1 of 112 µM and a K_0.5_ of 15–30 µM for MCT4 [18]–[20]. pCMBS at a concentration of 1 mM is expected to totally inhibit inward and outward lactate^-^ transport by MCT4 while also inhibiting not more than 90% of lactate^-^ flux through MCT1 [19], [22]. Similarly, [phloretin] of 1 mM is expected to completely inhibit lactate^-^ transport by MCT1, while also inhibiting not more than 90% of MCT4-mediated lactate^-^ transport [19], [22]. While these inhibitors do not exhibit high specificity for either isoform, they provide better separation than other inhibitors. It is also recognized that phloretin and pCMBS inhibit water, urea and chloride transport systems in various cell types, and may thus exert similar effects in skeletal muscle.

We hypothesized that skeletal muscle fibres exposed to an increased osmolarity, effected by increased extracellular [Na^+^] and [lactate^-^], would show an augmented (both larger and faster) RVI response compared to muscles exposed to increased osmolarity using NaCl or sucrose alone. The basis for the augmented response was hypothesized to be due to the rapid inward flux of lactate^-^, facilitated primarily by MCT1.

We report here that in the presence of increased extracellular osmolarity and [lactate^-^], single mouse skeletal muscle fibres exhibited a reduced volume loss, an augmented RVI response, and an RVI that comprised NKCC and MCT components compared to increased [NaCl]-treated myofibres. Also, preferential inhibition of MCT4 accelerated volume recovery. Bumetanide, which inhibits NKCC activity, had no effect on MCT-mediated lactate^-^ influx and RVI. Taken together, this study demonstrates, for the first time, that both NKCC and MCT1 are involved in volume regulation by non-contracting skeletal muscle fibres; a set of findings that significantly improves our understanding of skeletal muscle volume regulation [25].

## Methods

### Ethics approval

The animal handling and experiments were performed in accordance with the guidelines of the Canadian Council on Animal Care and with the approval of the University of Guelph's Animal Care Committee. Adult (2–4 months old) male and female C57bl/6 mice (Charles River Labs, Quebec, Canada) were housed at the University of Guelph in light and temperature controlled quarters. Food and water were provided ad libitum. Prior to removal of extensor digitorum longus (EDL) and peroneus muscles, each mouse was anesthetized by acute exposure to increasing ambient CO_2_ and humanely killed by cervical disarticulation when unconscious.

### Muscle fibres

Genders of mice were used at random, with each experimental series having or approaching a balance between male and female mice. Intact, isolated single muscle fibres from mouse EDL and peroneus muscles were used [5], [26], [27]. These hindlimb skeletal muscles express NKCC1 [23], MCT1 and MCT4 [14], [24].

This muscle fibre preparation is used in a number of experimental settings where fibre viability and an absence of metabolic rundown are important [5], [27]–[29]. These muscles were chosen because: (a) they are in close proximity to one another [anterior (EDL) and lateral compartments (peroneus)]; (b) the presence of long tendons facilitates their isolation and maintains their viability; and (3) they are small in diameter making their digestion in collagenase approximately the same duration. We therefore used a mixture of EDL and peroneus muscles; the EDL is comprised of 49% Type IIB and 51% Type IIA fibers, the peroneus longus is 48% Type IIB, 49% Type IIA and 3% Type I and the peroneus brevis is 58% Type IIB, 42% Type IIA and 1% Type I fibres [30].

Briefly, intact muscles were placed in a sterile-filtered collagenase solution (0.2% w/v Type I collagenase, Sigma-Aldrich, Mississauga, ON, Canada) in DMEM (Dulbecco's Modified Eagle Medium; Invitrogen, Burlington, ON, Canada). Following collagenase digestion, single fibres were obtained by repeated trituration of fibre bundles until many single fibres were visible. The wells of a 6-well plate were treated with 10% Matrigel (BD Biosciences, Mississauga, ON, Canada) in low glucose DMEM for 1 minute, rinsed with PBS (Invitrogen) and refilled with 1.5 ml of low glucose plating media. Plating media consisted of low glucose DMEM with 10% horse serum (Sigma-Aldrich), 0.5% chick embryo extract (MP Biomedicals) and passed through a 0.2 µm nylon filter. Four to six single fibres were placed into each of two wells and the plate placed in an incubator (5% CO_2_, 95% O_2_) for at least 60 minutes at 37°C in order to allow time for fibres to adhere to the Matrigel.

Plates containing fibres were taken to the microscopy suite, placed onto the stage of a Nikon Eclipse TE2000U inverted microscope (Melville, NY, U.S.A.) maintained at room temperature (21±1°C) for the duration of the experiments. Fibre position (x-y-z coordinates) from two locations per fibre, for each of up to three fibres, were programmed into software that allowed sequential movement between landmarks at fixed intervals. If fibres moved or displayed observable signs of distress (e.g. membrane blebbing), then that fibre was removed from the analysis. Digital images were obtained, using full spectrum light, of each landmark at 6 to 12 s intervals for a duration of up to 60 minutes.

Three cycles of baseline images were collected, then extracellular osmolarity was raised by up to 35% (∼100 mosmol/L) by adding the appropriate volume of 1 M NaCl, 1 M sucrose or 1 M NaLac to the well, followed by gentle swirling. This procedure required 15–20 s and image collection was re-initiated 30 s after addition of solute. Each fibre was imaged in two distinct microscope fields, and each of these was quantified at two locations for fibre width. The width measures for each image were obtained as far apart as possible (120 to 170 µm) within a relatively straight section of the fibre within the field of view. Depending on fibre orientation, between 150 and 200 µm of fibre was present within the field of view. No measurements were obtained less than 200 µm from a fibre end.

### Inhibition of NKCC or MCT

Transport inhibition experiments were conducted using bumetanide to inhibit the NKCC, phloretin to inhibit primarily lactate^-^ influx through MCT1, pCMBS to primarily prevent efflux of lactate^-^ that had entered fibres and secondarily to prevent influx through MCT4. Experiments simultaneously inhibiting NKCC and MCT were also performed. Inhibitors were dissolved in 100% dimethylsulfoxide (DMSO) to make a stock solution of each. Thirty (30) µL of 50 mM stock bumetanide were added to the individual wells containing 1.5 ml plating medium to achieve a final concentration of 1 mM; this resulted in a final DMSO concentration of 2% v/v. In experiments with either phloretin or pCMBS, 15 µL of 100 mM stock was added to the well to induce a final concentration of 1 mM; this resulted in a final DMSO concentration of 1% v/v. A combination of bumetanide with either phloretin or pCMBS results in a final DMSO concentration of 3% v/v.

Fibres were incubated in the presence of inhibitors for 30 minutes, during which fibres were landmarked for data acquisition. Baseline images of each landmarked fibre were taken three times prior to beginning the treatment protocol. Following the inhibitor incubation period, hypertonic challenge was administered by adding 1 M NaCl, sucrose or NaLac stock solution to increase extracellular osmolarity by the desired amount. Immediately following addition of solute, the image sequence was initiated and sequential imaging of each landmarked fibre was performed for up to 60 minutes.

### Experiments examining effect of DMSO on volume responses

Because DMSO was used as a solvent for the inhibitory solutions, experiments were carried out to investigate the response of muscle fibres to increased extracellular [DMSO] up to 3% v/v. Muscle fibres were plated and landmarked, and following the collection of baseline images appropriate volumes of DMSO were applied to mimic the final DMSO concentrations present in the transport inhibition treatments. Protocols administered with final DMSO of 2% and 3% were utilized resembling the upper concentrations reached in the presence of either single or dual transporter inhibition experiments. Following the 45 minute collection period, the fibres immediately underwent a second treatment, where extracellular osmolarity was increased ∼57 mosmol/kg by addition of NaCl. Images were immediately collected at 10 s intervals for up 60 minutes.

### Statistics

The volume responses were analyzed using a series of two-way ANOVAs with respect to time and treatment. Standardization of time points across all experiments could not be achieved because we obtained data from up to two sites on each of up to 3 fibres within a well. The number of fibres within a well that met criteria for use ranged from 1 to 3, resulting in variability for the time course of data acquisition, as well as variability in the number of muscles, fibres and fibre sites used for the final analysis. For some experiments the time point data for statistical analysis were obtained from each site of each fibre by interpolation between existing time points so as to match up time points across experiments that were statistically compared. When a significant F ratio was obtained, a one-way ANOVA with Holm-Sidak post-hoc test was performed to identify differences between means. Significance was accepted at p≤0.05. Data are reported as mean ± SE of the original, measured values as much as possible and minimized the use of interpolated values.

## Results

### Increased extracellular osmolarity causes cell volume loss and RVI

We first aimed to describe the dose – response characteristics of myofibres to increased extracellular osmolarity using sucrose, NaCl and NaLac. Raising extracellular osmolarity using sucrose or NaCl may have differing effects on the initial cell volume loss and subsequent RVI because of the differences in sarcolemmal permeability and conductance to sucrose compared to Cl^-^. The sarcolemma has a selected and regulated permeability to different electrolytes. Permeability to Na^+^ is low compared to Cl^-^ [31], [32] whereas Cl^-^ has a high membrane permeability and conductance due to the high density of voltage-sensitive Cl^-^ channels [33], [34]. Sarcolemmal permeability to lactate^-^ is high and mediated by transport through MCTs, primarily the low Km MCT1 [12], [15]. However increasing extracellular [lactate^-^] results in a significant decrease in membrane Cl^-^ conductance [34]. In contrast, sucrose is impermeant, such that an RVI can only be mediated by solute transport mechanisms and not equilibration of sucrose into cells.

**Figure 2:**
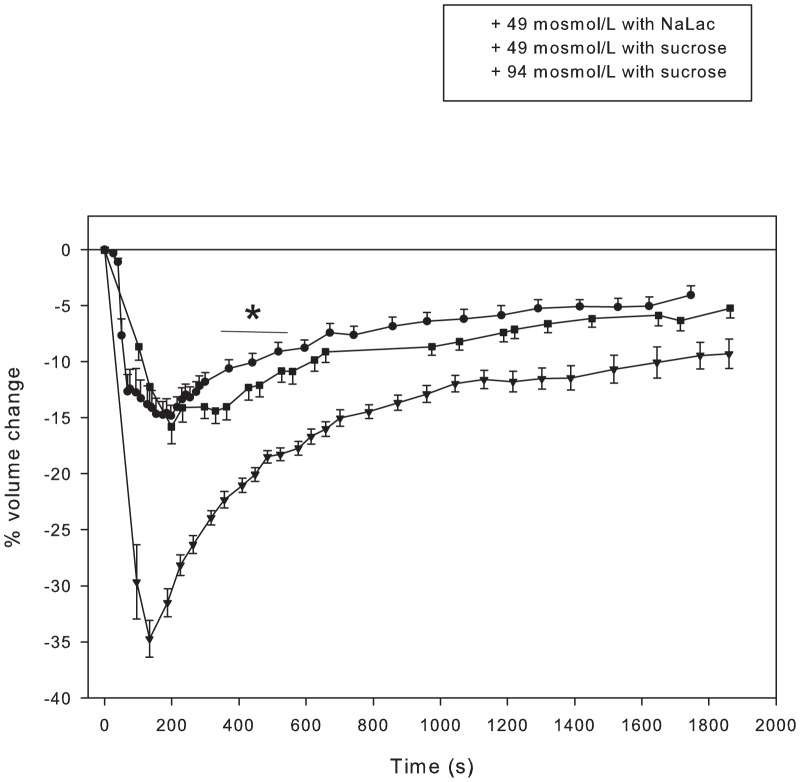
Cell volume loss with subsequent volume recovery in response to increased NaCl or sucrose. Time course of volume changes in single fibres where extracellular osmolarity was raised 49/L by addition of NaCl (•, n = 6 fibres from 4 muscles from 2 mice) or sucrose (▪, n = 8 fibres from 4 muscles from 2 mice), or by raising osmolarity by 94 mosmol/L using sucrose (▾ n = 11 fibres from 6 muscles from 3 mice). * The initial rate of volume loss and RVI were greater when NaCl was used to raise osmolarity compared to sucrose.

Increasing osmolarity by ∼58 mosmol/L using NaCl or sucrose resulted in identical volume losses, with peak volume loss occurring within 200 s (Fig. 2). The peak volume loss (∼15%) was similar to that predicted on the basis of increased extracellular osmolarity (17.7%). There was no difference in the rate of volume loss, however this is affected by passive mixing of added hypertonic solutions within the vicinity of the fibres. As expected on the basis of Cl^-^ versus sucrose permeability, early volume recovery with NaCl was significantly increased compared to sucrose. In summary, increased osmolarity resulted in an initial volume loss and an RVI due to increased NKCC activity [5], [35], [36] and equilibration of Cl^-^ across the sarcolemma according to electrochemical driving forces [34], [37].

Increasing extracellular osmolarity by ∼100 mosmol/L, by raising [sucrose], doubled the volume loss, and increased the rate of volume loss, compared to that seen with a 58 mosmol/L increase in osmolarity (Fig. 2). The increased volume loss also resulted in a more rapid initial (up to time point 500 s) volume recovery, with no difference in the slope of the RVI responses in the 2^nd^ slow phase of volume recovery. If muscle cells behaved as perfect osmometers, i.e. in the absence of a volume recovery mechanism, the peak volume loss to 100 mosmol/L and 58 mosmol/L increases in osmolarity should be 34% and 18%, respectively, and the cells would remain in a shrunken state. The data from Fig. 2 illustrates that the initial muscle volume loss depends primarily on the increase in extracellular osmolarity when these solutes are used. The muscle cells did not remain shrunken and thus did not behave as perfect osmometers, and the rate of volume recovery was dependent on extracellular osmolarity and the solute used.

### Increased extracellular osmolarity using lactate^-^ attenuates volume loss

**Figure 3:**
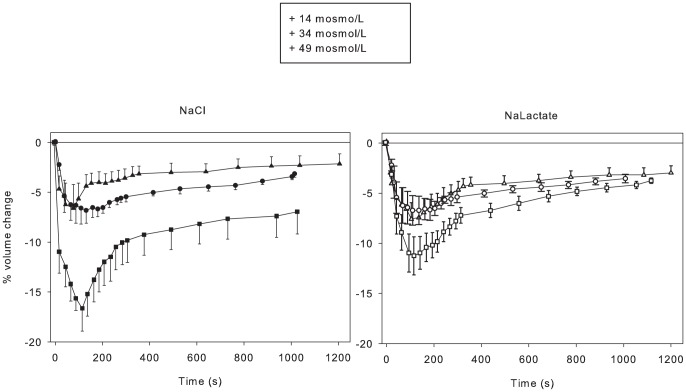
Osmolarity dependence of volume loss and attenuated volume loss with elevated [NaLac] compared to NaCl. Time course of volume changes in single fibres where extracellular osmolarity was raised by 14 (▴), 34 (•) and 49 mosmol/L (▪) by raising [NaCl] or [NaLac]. NaCl + 49 mosmol/L: n = 6 fibres from 4 muscles from 2 mice; NaLac + 49 mosmol/L: n = 30 fibres from 16 muscles from 8 mice). NaCl + 34 mosmol/L: n = 14 fibres from 7 muscles from 3 mice; NaLac + 34 mosmol/L: n = 14 fibres from 7 muscles from 3 mice or 7.5 mM NaCl. NaCl + 14 mosmol/L: n = 20 fibres from 6 muscles from 3 mice; NaLac + 14 mosmol/L: n = 12 fibres from 6 muscles from 3 mice.

Marked increases in extracellular [lactate^-^] occur with high intensity exercise, and we were interested to determine if non-contracting muscle cells responded differently to increased [NaLac] than to increased [NaCl]. When increases of extracellular [NaCl] and [NaLac] were 7.5 mM or 18.5 mM, the cell volume losses were similar between concentrations and solutes (Fig. 3). A higher extracellular [lactate^-^] increase of 30 mM was also used because this has been shown to occur in vivo with high intensity exercise [4]. With increases in extracellular [NaLac] and [NaCl] of 30 mM, the rate and duration of volume loss were greater for NaCl than for NaLac. The initial phase of volume recovery was also more rapid in response to the raised NaCl. These findings demonstrate that the rate and magnitude of the volume responses are dependent on anion permeability of the sarcolemma when extracellular concentrations increase above ∼20 mM. The entry of lactate^-^ is more rapid than for Cl^-^ during the volume loss period, and the rapid lactate^-^ entry attenuated the rate, duration and magnitude of volume loss compared to that seen with raised NaCl. It is postulated that the rapid entry of lactate^-^ is facilitated by MCTs, primarily MCT1.

### Inhibitors of solute transport

#### NKCC inhibition

**Figure 4:**
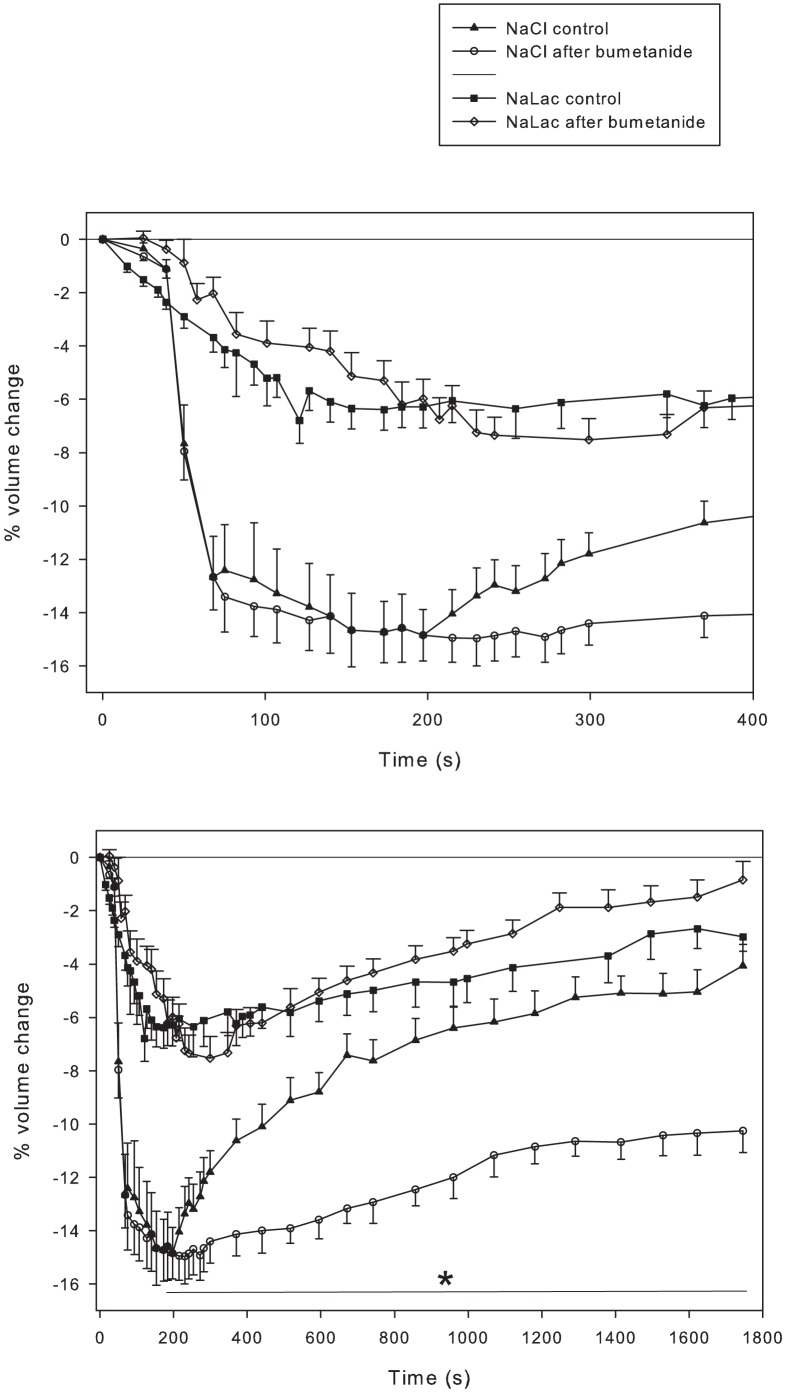
NKCC inhibition does not affect muscle volume responses when osmolarity is raised using NaLac. Time course of volume changes in single fibres to raised osmolarity (+49 mosmol/L) using NaCl or NaLac, in the absence or presence of NKCC activity inhibition using 1 mM bumetanide. NaLac controls (▪, n = 39 fibres from 21 muscles from 6 mice) compared to NKCC inhibition (⋄; n = 30 fibres from 16 muscles from 8 mice). NaCl controls (▴; n = 13 fibres from 6 muscles from 4 mice) compared to NKCC inhibition (○; n = 16 fibres from 6 muscles from 3 mice). The top panel expands the first 400 s. * significantly lower than NaCl control.

Bumetanide (1 mM) was used to inhibit NKCC activity in the presence of raised NaCl and raised NaLac. When osmolarity was raised using NaCl, fibres with inhibited NKCC exhibited a greater volume loss and a slow, incomplete volume recovery (Fig. 4). In contrast, when osmolarity was raised using NaLac, fibres with inhibited NKCC showed a similar magnitude, but slowed, volume loss compared to NaLac control fibres; there was no difference in volume recovery. Therefore, when extracellular osmolarity is raised using NaLac, RVI occurred without significant activation of the NKCC, implicating a role for lactate^-^ influx through MCTs. Therefore the RVI response does not depend solely on the function of the NKCC, illustrating that skeletal muscle has an NKCC-independent means of eliciting volume increases.

#### MCTs inhibition

The aforementioned experiment with raised extracellular [NaLac] indicated a high membrane permeability to lactate^-^ and a high rate of net lactate^-^ flux into fibres during the volume loss phase, and that RVI occurred even with NKCC inhibition. We tested the hypothesis that rapid lactate^-^ influx via MCTs was responsible for the attenuated volume loss and RVI when extracellular [NaLac] was raised. Phloretin (1 mM) should completely inhibit lactate^-^ influx by MCT1 and inhibit ∼90% of influx by MCT4, while pCMBS (1 mM) should completely inhibit influx by MCT4 and inhibit ∼90% of influx by MCT1 [19], [22]. The effects of MCT inhibition were compared to that of NKCC inhibition using bumetanide.

**Figure 5:**
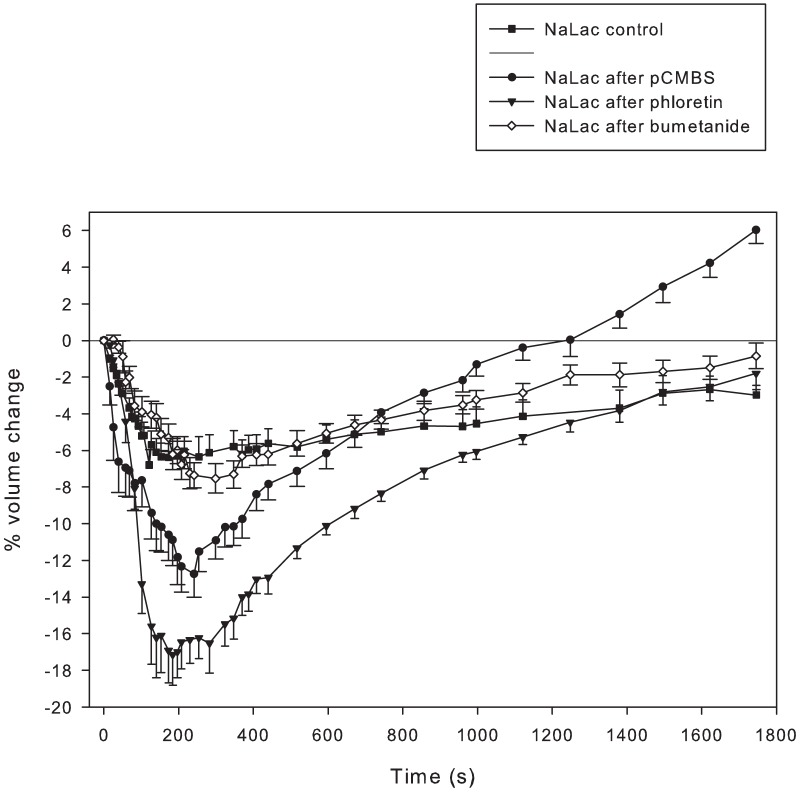
MCT inhibition increases the magnitude of volume loss and influences the RVI response. Time course of volume changes in single fibres where MCT activity was inhibited using either phloretin (▾, n = 7 fibres from 4 muscles from 2 mice) or pCMBS (•, n = 11 fibres from 6 muscles from 3 mice), or where NKCC activity was inhibited using bumetanide (⋄, n = 39 fibres from 21 muscles from 6 mice) compared to control (▪; n = 30 fibres from 16 muscles from 8 mice). Fibres were incubated in the presence of inhibitor for 30 minutes prior to addition of NaLac to raise extracellular osmolarity by 49 mosmol/kg. pCMBS resulted in a greater volume loss and more rapid volume recovery than seen in controls or in bumetanide treated fibres. Phloretin resulted in a greater volume loss than seen in controls, bumetanide treated fibres and pCMBS treated fibers. The rates of volume recovery in pCMBS and phloretin treated fibres were similar.

In these experiments, [NaLac] or [NaCl] was increased by ∼25 mM which raised extracellular osmolarity by ∼49 mosmol/L. As can be seen in Fig. 5, inhibition of lactate^-^ influx (by phloretin) resulted in a doubling of the magnitude and rate of cell volume loss compared to the NaLac control trial, such that the time at which peak volume loss occurred was the same (Fig. 5). This was followed by a relatively prolonged (∼150 s) nadir and then a rapid and complete RVI, requiring 1800 s, with no overshoot. This volume response, in the presence of MCT inhibition, was very similar to that of NaCl in the absence of transport inhibition (see Fig. 3), indicating that the NKCC was responsible for the RVI in these phloretin-treated fibres. The similarity of responses between these two trials also indicates complete inhibition of lactate^-^ entry when phloretin was used.

When pCMBS was used to inhibit lactate^-^ flux, there was a doubling of volume loss compared to the NaLac control trial, but a reduced and slowed volume loss compared to phloretin trial (Fig. 5). Also differing from both the NaLac control and phloretin trials, pCMBS resulted in a more rapid and sustained RVI that produced restoration of initial cell volume within ∼1200 s, and a volume overshoot to 6% above initial cell volume during the final 600 s of the experiment. pCMBS, compared to phloretin, thus appeared to have two main effects: 1) pCMBS did not fully inhibit lactate^-^ entry into the myofibres and [2] the more rapid volume recovery and volume overshoot indicated that backflux of lactate^-^ out of the cell was also inhibited allowing for a more rapid and sustained accumulation of solute inside pCMBS treated fibres.

#### Inhibition of both MCT and NKCC

**Figure 6:**
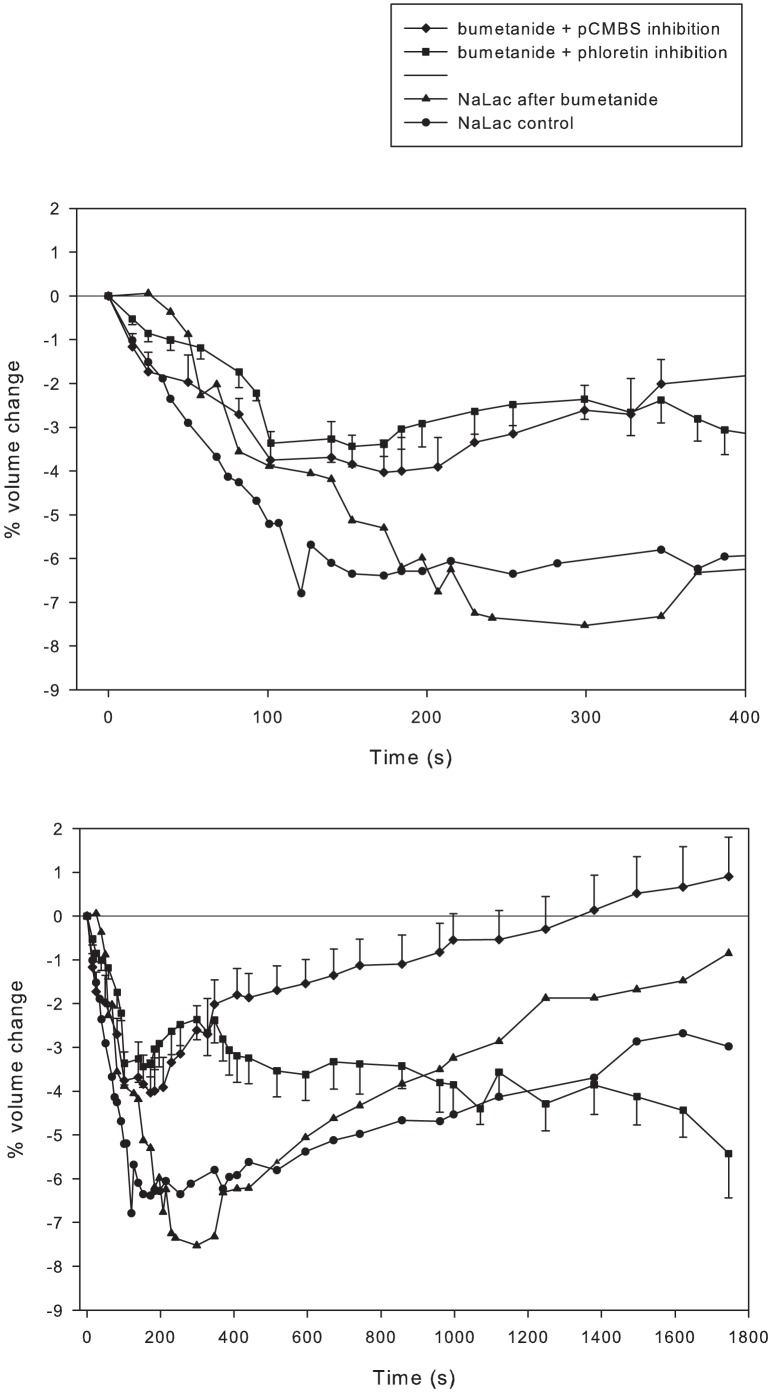
Combined inhibition of MCT and NKCC minimizes volume loss and associated RVI. Time course of volume changes in single fibres where NKCC and monocarboxylate transport activity was inhibited using bumetanide with phloretin (▪ n = 19 fibres from 8 muscles from 4 mice) or bumetanide with pCMBS (<$>\raster(130%)="rg1"<$> n = 13 fibres from 7 muscles from 4 mice) compared to NaLac control (• n = 30 fibres from 16 muscles from 8 mice) and NaLac after NKCC inhibition (▴ n = 39 fibres from 21 muscles from 6 mice). Fibres were incubated in the presence of inhibitors for 30 minutes prior to addition of NaLac to raise extracellular osmolarity by 57 mosmol/kg. The top panel expands the first 400 s.

The results of the MCT inhibition experiments suggested a normal activation of the NKCC when fibres were exposed to increased NaLac. Therefore, it was hypothesized that blocking both the NKCC and MCT together should result in a greater magnitude of volume loss and nearly complete inhibition of the transport-facilitated RVI responses. However, and in contrast to the hypothesis, combined inhibition of these transport proteins resulted in small and slow volume losses (Fig. 6) compared to inhibition with either bumetanide, pCMBS or phloretin alone (Fig. 5). This volume loss was also reduced compared to the NaLac control (Fig. 6). The rate of volume loss and the time to peak volume decrease were similar in both combined inhibitor conditions. The RVI response that occurred in response to the combined transporter inhibition conditions were biphasic, and different. In both conditions there was an initial RVI of about 200 s duration following the volume loss phase. Thereafter, inhibition with pCMBS and bumetanide resulted in a slower, though continuing, RVI to recovery of baseline volume at 1800 s. In contrast, with combined phloretin and bumetanide, fibres stopped gaining volume and lost a further 2–3% of volume during the remainder of the experiment.

### The effect of DMSO on cell volume responses

**Figure 7:**
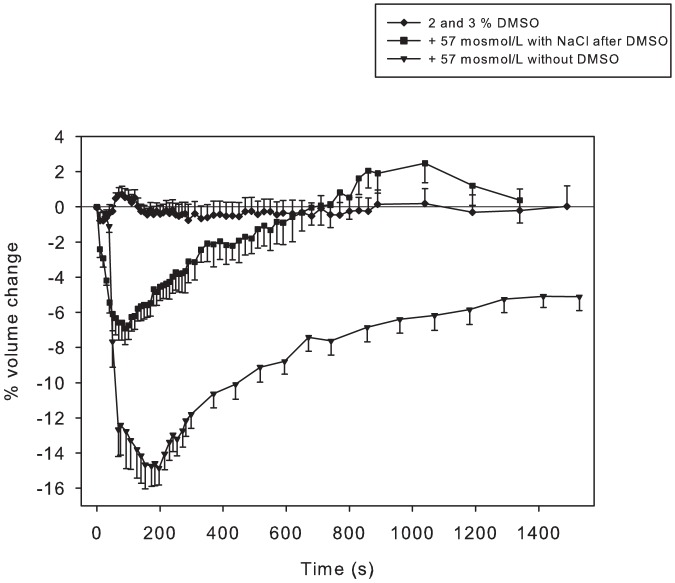
Time course of volume changes in single fibres treated with DMSO. Cell volume did not change after addition of DMSO to final concentration of 2 to 3% (<$>\raster(130%)="rg1"<$>) for 2700 s (45 minutes; no change after the 1500 s shown in the figure). At 45 minutes these DMSO-treated fibres experienced a 57 mosmol/L increase in extracellular osmolarity using NaCl (▪; time was re-set to 0 s). The response of fibres not treated with DMSO to increased osmolarity (▾ 57 mosmol/L using NaCl; data from Fig. 1) is shown for comparison. DMSO treatment: n = 16 fibres from 8 muscles from 3 mice. NaCl treatment: n = 12 fibres from 6 muscles from 3 mice.

DMSO was used to solubilize and produce high-concentration stock solutions of the transport inhibitors. DMSO is also osmotically active and can result in altered membrane permeability to solutes and water. We therefore investigated the potential effects of DMSO on the cell volume responses. Further, we also determined if initial exposure of cells to DMSO affected subsequent responses to increased extracellular osmolarity. Cells treated with DMSO (2–3%) exhibited negligible volume loss (1%) followed by immediate recovery, non-significant volume overshoot and a subsequent return to baseline (Fig.7). When these fibres were subsequently exposed to a 57 mosmol/l increase in osmolarity with 30 mM NaCl, there was a 7% volume loss, significantly less than the 15% volume loss seen in fibres not pre-treated with DMSO.

## Discussion

The present study demonstrated that mouse skeletal muscle fibres responded to increased extracellular osmolarity by initially shrinking then regaining volume using sarcolemmal NKCC- and MCT-mediated transport mechanisms. We further demonstrated that, when extracellular [lactate^-^] and osmolarity were elevated, lactate^-^ influx by MCTs contributed to a diminished volume loss and to the volume recovery. Importantly, the inwardly-directed lactate^-^ flux through MCT1 appeared to be primarily responsible for these effects and occurred in association with increased NKCC activity. Inhibition of the primarily outwardly-directed lactate^-^ flux through MCT4 appeared to prevent lactate^-^ back-flux from cells and resulted in a more rapid recovery of volume, and an increase in cell volume above baseline.

Taken together, these experiments define, for the first time, the underlying mechanisms of how volume regulation within non-contracting skeletal muscle may influence circulating blood volume during and following high-intensity exercise.

### Sucrose versus NaCl

The rapid and osmotically complete initial decrease in cell volume seen with increased extracellular NaCl and sucrose indicates an absence of net solute and water influx during the rapid phase of fluid loss. The assumed intracellular osmolarity of undisturbed fibres is 328 mosmol/kg (equal to that of the DMEM solution). The osmotic coefficients of NaCl and sucrose are both close to 0.94 [37], and raising extracellular [sucrose] by 100 mM increases osmolarity by 94 mosmol/kg. Raising extracellular osmolarity by 94 mosmol/kg by addition of sucrose or NaCl, in the absence of any volume recovery mechanism and assuming that all cell water participates osmotically, should result in a 33% decrease in cell volume. This is identical to the initial cell volume loss seen during the 100 mM sucrose trial (Fig. 2). The decreases in cell volume in response to 60 mM sucrose and 30 mM NaLac were also similar to predicted (Fig. 2) and also exhibited a concentration-dependent effect of increased extracellular osmolarity on the magnitude of cell volume loss and RVI response, similar to previously reported in intact muscle [5].

It appears that in both the sucrose and NaCl trials that NKCC activity was not stimulated until close to the time at which peak volume loss occurred, and that there was no (or minimal) increase in the passive entry of Na^+^, K^+^ or Cl^−^ through ion channels. Because sucrose is membrane impermeant, sucrose could not participate in the subsequent volume recovery. Volume recovery under conditions of increased extracellular [sucrose] is highly bumetanide-sensitive, and thus, mainly due to increased NKCC activity [5], [35], [36]. Despite high membrane permeability to Cl^−^ [31], [37], a net influx of Cl^−^ through high conductance Cl^−^ channels [ClC1; 34] did not appear to have occurred, arguing against a rapid Cl^−^ equilibration across the sarcolemma and T-tubule system. This must be because Cl^−^ channel conductance decreased somewhat with increased extracellular osmolarity, perhaps associated with the modest membrane depolarization that occurs when muscle is exposed to hypertonic solutions: -10 mv for 50 mosmol/kg increase in osmolarity [39], [40].

### Roles of MCTs in cell volume regulation

Volume losses seen in response to raised NaCl and sucrose were similar when extracellular osmolarity was raised by the same amount. In contrast, when [NaLac] was increased by 30 mM, the cellular volume loss was only about half of that seen with equi-osmolar increases using NaCl or sucrose. As the sarcolemma is highly permeable to lactate^-^ due to the presence of MCTs [22], we propose that the reduced rate and magnitude of volume loss in the NaLac-exposed fibres was due to appreciable uptake of lactate^-^ by the fibres during the first 180 s (Fig. 3); a finding consistent with previous reports [41] illustrating a net lactate^-^ influx during the initial minutes following exposure to increased [lactate^-^].

We tested the hypothesis that rapid lactate- influx contributed to reduced volume loss when extracellular [NaLac] was increased. Using phloretin to inhibit MCT1 in fibres exposed to elevated NaLac produced a volume loss ∼2-fold greater than that seen with raised extracellular NaLac in the absence of transport inhibitors. The rate and magnitude of volume loss was very similar to that seen in uninhibited fibres exposed to NaCl or sucrose (Fig. 4). This increased volume loss in the presence of phloretin indicates that in the presence of raised extracellular [lactate^-^] there is a marked, rapid influx of lactate^-^ through MCT1 (primarily) and that the rapidity of this solute influx reduces the volume loss associated with the increase in extracellular osmolarity. Because it is highly unlikely that there was significant influx of lactate^-^ via the MCT4 in this condition, the resultant RVI response strongly indicates that the NKCC is also activated under these conditions, and that the ensuing volume recovery occurred primarily by solute influx via the NKCC. Indeed, the time course of volume loss and recovery of phloretin-treated fibres in the presence of raised [NaLac] was very similar to that of untreated fibres exposed to an elevated [NaCl].

Under conditions of raised extracellular [lactate^-^], net lactate^-^ uptake occurs faster than its rate of oxidation [42] and intracellular [lactate^-^] increases exponentially, reaching an equilibrium concentration 3–4 mM less than the extracellular concentration in ∼30 minutes [41], [43]. This raises the possibility of an increasing backflux of lactate^-^ via MCT4 from the fibres over time. A contribution of inward flux of lactate^-^ through MCT4 was examined using 1 mM pCMBS which totally inhibits lactate^-^ flux through MCT4 but only inhibits ∼90 of flux through MCT1 [22]. As predicted from the 30 mM higher lactate^-^ Km for MCT4 than for MCT1, pCMBS resulted in a volume loss intermediate to that of the NaLac control and the NaLac-phloretin treatment (Fig. 4). This result suggests that despite significant (∼90%) inhibition of MCT1-mediated lactate^-^ flux there still occurred appreciable net lactate^-^ influx into fibres during the volume loss phase. The unexpected findings of this experiment were the rapidity of the RVI (30% faster than in the NaCl and sucrose conditions) and the volume overshoot that occurred once the baseline volume had been reached. Because 1 mM pCMBS should completely inhibit MCT4, no appreciable backflux of accumulated lactate^-^ through this pathway should occur, resulting in a more rapid accumulation of lactate^-^ inside the fibres.

The volume overshoot observed in this condition was likely the result of the combination enhanced lactate^-^ accumulation and an increased NKCC activity. It remains unknown why NKCC activity would not be more rapidly down-regulated under these conditions, thus preventing or minimizing the volume overshoot. These data, therefore, also suggest that the absence of volume overshoot seen in the NaLac control and phloretin treatments may have been partially due to some backflux of lactate^-^ through the MCTs.

### Combined NKCC and MCT inhibition

It appeared that the NKCC was activated and fully responsible for the RVI under conditions in which extracellular [NaLac] was raised in the presence of MCT inhibition. We therefore hypothesized that combined inhibition of NKCC and MCT activities should result in an initial volume loss (similar to that seen with phloretin) and abolish the RVI. This was tested by incubating fibres with a combination of bumetanide with either phloretin or pCMBS. In contrast to the hypothesis, both of these combinations resulted in an ∼70% attenuation of volume loss such that the initial volume loss was only 3–4.5% of cell volume (Fig. 5). Though we would surmise that these effects cannot be completely attributed to the use of DMSO as a solvent (∼3% volume loss; Fig. 7), it can not be ruled out that the combination of inhibitors and DMSO exerted effects different from the inhibitors without DMSO. Future studies using alternative approaches (genetic, silencing RNA, etc.) will help to clarify this matter.

When one considers the effects to be independent of DMSO, the effects of combined MCT and NKCC inhibition may be interpreted as follows. The minimal loss of cell volume may result from reduced membrane water permeability, increased membrane permeability of solute, or a combination of the two. With respect to membrane ion permeability, it is likely that Cl^−^ conductance was markedly reduced [39], [40], leaving roles for increased permeability to Na^+^ and/or K^+^. The result could be explained by a rapid, passive K^+^ loss through various K^+^ channels that contribute to muscle K^+^ balance [44]. The effect could also be caused by an increased passive Na^+^ entry – the most likely mechanism would be through voltage-gated Na^+^ channels. Both of these mechanisms imply that treatment of muscle with bumetanide and an MCT inhibitor, in the presence of elevated osmolarity and [lactate^-^] produced a pronounced membrane depolarization that opened voltage-gated Na^+^ channels and K^+^ channels. While these possibilities could be tested experimentally, the contribution of these mechanisms to cell volume regulation in mouse muscle cells would be of minor significance.

### Use of DMSO to solubilize transport inhibitors: effect on muscle volume responses

In the present study we provide evidence of the effects of DMSO on cell volume responses in single fibres. We landmarked single fibres and obtained a series of baseline images. We then added DMSO to increase final solution [DMSO] by 2% (282 mM) and 3% (423 mM) and images were obtained at 10 s to 30 s intervals for 45 minutes; there were no differences between these two treatments. If DMSO even transiently increased extracellular osmolarity, as has been suggested [45], one would predict a decrease in cell volume. In contrast, we observed a small, non-significant and briefly transient increase in cell volume (Fig. 7). At the end of the 45 minute period, extracellular osmolarity was increased ∼57 mosmol/L by the addition of NaCl.

We observed a mean volume loss of 7%, compared to 15% in the control experiments, as well as a complete RVI response (Fig. 7). Therefore, pre-treatment of muscle fibres for 45 minutes with [DMSO] up to 3% attenuated, but did not abolish, the initial volume loss and had no effect on RVI. This result is similar to that observed in rat renal brush border membrane vesicles, where DMSO at concentrations up to 400 mM decreased the magnitude, but not the rate constant, of osmotic volume loss [46]. The results may be interpreted in two ways: (a) treatment of fibres with [DMSO] of 3% for 45 minutes can affect cell volume by transiently or partially increased membrane permeability to solutes, thus reducing the volume loss; or (b) DMSO may have reduced membrane permeability to water flux, such that net loss of water from cells is reduced and RVI maintained. Furthermore, and if an effect of DMSO is to reduce water flux through aquaporins [46], the overall impact of DMSO on aquaporins may be physiologically not important because Yang et al. [47] provided evidence in mouse muscle fibres that aquaporins do not contribute significantly to plasma membrane water permeability.

While elucidation of the mechanism(s) of actions of DMSO on the membrane ion and water permeability requires additional experiments, it can be stated that at the end of the 45 minute treatment period that DMSO would be equilibrated across the membrane. Thus, the experiments described herein indicate that DMSO had a negligible effect on membrane water permeability.

In the transport inhibitor experiments in which fibres were treated (for 45 minutes) with DMSO containing bumetanide, or phloretin, or pCMBS we did not see a reduced volume loss when extracellular osmolarity was raised. In contrast, we always observed an increased volume loss. This result is inconsistent with DMSO reducing membrane permeability to solute. The magnitude of volume loss with bumetanide and raised NaCl agreed with a complete, initial impairment of NKCC activity and achievement of a rapid osmotic equilibrium with the extracellular solution, i.e. cells initially behaved as perfect osmometers. This appears to be a clear bumetanide effect and not a DMSO effect. Fibres also slowly recovered partial volume indicating secondary mechanisms of volume recovery.

Taken together, the transport inhibitor and DMSO experiments demonstrate that the use of DMSO as a solvent and vehicle to apply the inhibitory drugs (bumetanide, phloretin, pCMBS) to the media does not compromise the integrity of volume loss and resultant volume recovery due to osmotic challenge.

### Physiological rationale for skeletal muscle volume regulation

With high intensity exercise, plasma osmolarity can increase by 35 mosmol/L due to increases in plasma [lactate^-^], [Na^+^], [Cl^-^] and [K^+^] [see 48]. Within contracting muscles, at the onset of exercise, there occurs rapid hydrolysis of phosphocreatine and the production of two osmotically active molecules: creatine and inorganic phosphate. At the same time, glycogenolysis results in the production of lactate^-^, another osmolyte. The increase in intracellular osmolarity of contracting muscle during the first few minutes of exercise results in the net flux of ion-poor fluid into contracting muscles from the plasma and interstitial fluid compartments [2], [3]. This fluid loss from plasma, along with increases in [K^+^] and [lactate^-^] from contracting muscle, is largely responsible for the increase in plasma osmolarity. This increase in plasma osmolarity results in the osmotic flux of water from non-contracting tissues into the vascular compartment, and causes non-contracting cells to lose volume [49]. It is this cell shrinkage that was mimicked in the present study. Non-contracting muscle cells osmotically lose fluid when extracellular osmolarity is raised, however our results indicate that this does not appear to be well tolerated, and the skeletal muscle cells that are caused to osmotically shrink respond by activating ion transport mechanisms that serve to restore cell volume. We further demonstrate that the mechanisms by which this occurs are by increased NKCC activity and by increased entry of lactate^-^, via MCT1.
